# USP32 Promotes Colorectal Carcinoma Progression Through Activating NF‐κB Signalling Pathway

**DOI:** 10.1111/jcmm.70457

**Published:** 2025-03-23

**Authors:** Xiaofan Duan, Gaoshaer Yeerkenbieke, Siping Huang, Yanjun Feng

**Affiliations:** ^1^ School of Medicine Tongji University Shanghai China; ^2^ Department of Oncology Shanghai GoBroad Cancer Hospital, China Pharmaceutical University Shanghai China; ^3^ Department of surgical oncology Shanghai GoBroad Cancer Hospital, China Pharmaceutical University Shanghai China; ^4^ Shanghai East Hospital, Nanjing Medical University Shanghai China; ^5^ Department of Oncology Shanghai Artemed Hospital Shanghai China

**Keywords:** colorectal carcinoma, immunotherapy, NF‐κB signalling pathway, tumour environment, USP32

## Abstract

Ubiquitin‐specific protease 32 (USP32) plays a key role in cancer progression. However, its functions in colorectal carcinoma (CRC) are still unexplored. In our study, we explored the expression and clinical significance of USP32 in CRC as well as its relationship with the tumour microenvironment (TME). As a result, we found that USP32 is overexpressed in CRC and it is associated with poor outcomes in CRC patients. In addition, the expression of USP32 is significantly related to the activation of the NF‐κB signalling pathway and the immune infiltrates of the TME. Wet experiments also confirmed that USP32 is critical for the proliferation, survival, and migration of CRC cells and tumour growth, which may be due to the activation of the NF‐κB signalling pathway. In conclusion, targeting the USP32–NF‐κB axis may be a novel treatment strategy for CRC patients.

## 1 Introduction

Colorectal cancer (CRC) is a significant form of cancer, ranking third in importance, which is an important threat to human health [1]. It is estimated that there will be approximately 190,000 new cases and 600,000 deaths from colon cancer in the United States in 2022 [2, 3, 4]. Globally, there were around 1.5 million new cases and 580000deaths caused by CRC in 2020 [3]. The first‐line treatment options for CRC are diverse. However, clinical limitations, such as resection evasion during surgery and insufficient treatment with chemotherapy, lead to a relatively low 5‐year survival rate of CRC patients [5, 6]. Therefore, there is an urgent need for the development of new diagnostic biomarkers and therapeutic agents to address this disease.

Ubiquitination is a post‐translational modification that is involved in protein degradation through the ubiquitin–proteasome system and plays a key role in several biological processes, including DNA transcription and repair, trafficking events, cell development, immune response and signal transduction [7]. Mechanistically, the ubiquitination process is catalysed by an enzyme complex (E1, E2 and E3 ligase enzymes). Conversely, deubiquitinases (DUBs) can remove ubiquitin from target ubiquitylated substrates, thus inhibiting this process [8]. Extensive research has demonstrated that DUBs act as critical regulators of signal pathways involved in different diseases [9, 10]. Therefore, investigating the function of DUBs and their downstream effectors will provide valuable insights into the molecular mechanisms underlying cancer initiation while guiding the development of novel therapies.

USPs were found to constitute the largest subfamily with over 60 members of DUBs. In recent years, the *USP32* gene, which maps to 17q23, has been found to be overexpressed in several kinds of cancers such as breast cancer [11], epithelial ovarian cancer [12], small cell lung cancer [13], glioblastoma [14], gastric cancer [15] and liver cancer [16]. Mechanistically, the abnormal activation of USP32 contributes to cancer cell proliferation, invasion and drug resistance via regulating the expression of downstream factors such as SMAD, SHMT2, SLC35F2 and FDFT1 [17]. Although the multiple functions of USP32 in cancer have been widely reported, no studies have investigated the expression and potential role of USP32 in CRC.

In our project, through performing bioinformatics analysis and wet experiments, we explored the expression, clinical significance and roles of USP32. Of note, we also revealed the associations between USP32 and CRC's TME and found that the NF‐κB signalling pathway may be downstream of USP32.

## 2 Materials and Methods

### 2.1 Microarray Data Analysis

The mRNA expression in TCGA‐COAD contains 355 CRC samples and 380 non‐cancer colon tissues, and the relevant clinical data were obtained. In addition, USP32 expression in CRC and non‐cancer colon tissues was also acquired from GSE18105, GSE71187, GSE87211 and GSE77953.

### 2.2 Immunohistochemistry Staining

The HPA database (https://www.proteinatlas.org/) was utilised to explore the protein levels of USP32 in CRC tumour tissues and colon normal tissues.

### 2.3 Cell Culture

CRC cell lines including HCT8, HCT116, HCT15, DLD1, SW620, SW480, RKO, MC38 and CT26 were purchased from ATCC. Cells were cultured in the DMEM/RPMI 1640 medium with 10% foetal bovine serum.

### 2.4 Survival Analysis

We used the Kaplan–Meier plotter (https://kmplot.com/analysis/) to explore the clinical significance of USP32 in CRC patients. Subsequently, the results underwent a log‐rank test.

### 2.5 Functional Enrichment Analysis

Gene set enrichment analysis (GSEA) was used to explore the biological processes and signal pathways associated with USP32 in CRC.

### 2.6 Immune Cell Infiltration Analysis

To investigate the association between USP32 expression and immune cell infiltration in CRC, we utilised the CAMOIP database and the quanTIseq algorithm [18, 19]. Specifically, we used the quanTIseq method to appraise NK cells, CD4 + and CD8 + T cells, Tregs, B cells, M1 and M2 macrophages, monocytes, neutrophils, dendritic cells and other immune cells. Concurrently, we also utilised the CAMOIP database to investigate the interaction between USP32 and immune scores including stromal fraction, intratumour heterogeneity, TIL regional fraction, proliferation, wound healing, macrophage regulation, fraction altered, aneuploidy score, homologous recombination defects, BCR evenness, lymphocyte infiltration signature score, IFN‐gamma response, TGF‐beta response, number of segments, BCR Shannon, BCR richness and CTA score in CRC.

### 2.7 Single‐Cell RNA‐Sequencing (scRNA‐Seq) Analysis

The Single Cell Portal database (https://singlecell.broadinstitute.org/single_cell/study/SCP1162) was used to investigate USP32's expression at single‐cell levels in a CRC single‐cell sequencing dataset [20].

### 2.8 Western Blot (WB) Analysis

WB analysis's procedure was performed according to our previous study [21]. Primary antibodies are as follows: anti‐USP32 (Cat No. 18838‐1‐AP, Proteintech), anti‐IKKα (Cat No. 61294, CST), anti‐IKKβ (Cat No. 8943, CST), anti‐p65 (Cat No. 8242, CST), anti‐Phospho p65 (Cat No. 13346, CST), anti‐GAPDH (Cat No. 2118, CST) and anti‐β‐actin (Cat No. 3700, CST).

### 2.9 Establishment of USP32 Overexpressed and Knockdown Cell Line

For USP32 overexpression, we constructed lentivirus CMV containing USP32 overexpression plasmids. For USP32 knockdown, lentivirus‐mediated transduction of shRNA targeting USP32 was also carried out. Two shRNA targeting sequences were 5′‐GGACAGUUAUAUGCACUUATT‐3′ (sh1) and 5′‐GACCUGUGGACUCUCAUAUTT‐3′ (sh2), respectively. Cells that stably expressed USP32 or shUSP32 were selected by applying puromycin.

### 2.10 Cell Function Assays

Cell Counting Kit‐8 (CCK8), transwell and colony formation assays were used to explore the oncogenic abilities of CRC cells, and the procedure was performed according to our previous study [21].

### 2.11 Flow Cytometry Analysis

Apoptotic cancer cells were detected using an Annexin V‐FITC kit (Dojindo Laboratories, Japan), and cell apoptosis rates were investigated by flow cytometry in triplicate (Arial III, BD Biosciences, CA, USA). For analysing infiltrating myeloid‐derived suppressor cells (MDSCs) and M2 macrophages in CT26 tumours, we cut tumours and digested them with collagenase D and DNase I. The suspensions were then filtered and blocked with anti‐mouse CD16/32. Finally, cells were stained with specific antibodies to identify MDSCs and M2 macrophages by utilising BD Biosciences (Arial III).

### 2.12 Animal Model

BALB/c nude mice aged 6 weeks and BALB/c mice aged 8 weeks were purchased from Charles River. DLD1‐vector, DLD1‐USP32, HCT116‐shNC, HCT116‐shUSP32‐1, and HCT116‐shUSP32‐2 cells were subcutaneously injected into each BALB/c nude mouse. CT26‐vector and CT26‐USP32 cells were subcutaneously injected into each BALB/c mouse. The mice were sacrificed, and the volume and weight of each tumour were measured. All animal experiments were approved by the Institutional Animal Care and Use Committee of Shanghai East Hospital.

### 2.13 Immunohistochemistry (IHC)

IHC analyses of tumours from DLD1 and HCT116 xenograft tumours were performed, and the procedure was performed according to our previous study [21]. Primary antibodies were anti‐IKKα (Cat No. A2062, ABclonal), anti‐IKKβ (Cat No. A2087, ABclonal), anti‐Phospho p65 (Cat No. ab86299, Abcam) and anti‐USP32 (Cat No. 18838‐1‐AP, Proteintech).

### 2.14 Statistical Analysis

Graphing and statistical analysis were performed by using GraphPad Prism. An unpaired two‐tailed Student's *t*‐test/Mann–Whitney–Wilcox test was performed to analyse the statistical significance. Statistically significant results were identified by a *p*‐value < 0.05.

## 3 Results

### 3.1 USP32 Expression Is Increased in CRC and Associated With Clinical Outcomes in CRC Patients

**FIGURE 1:**
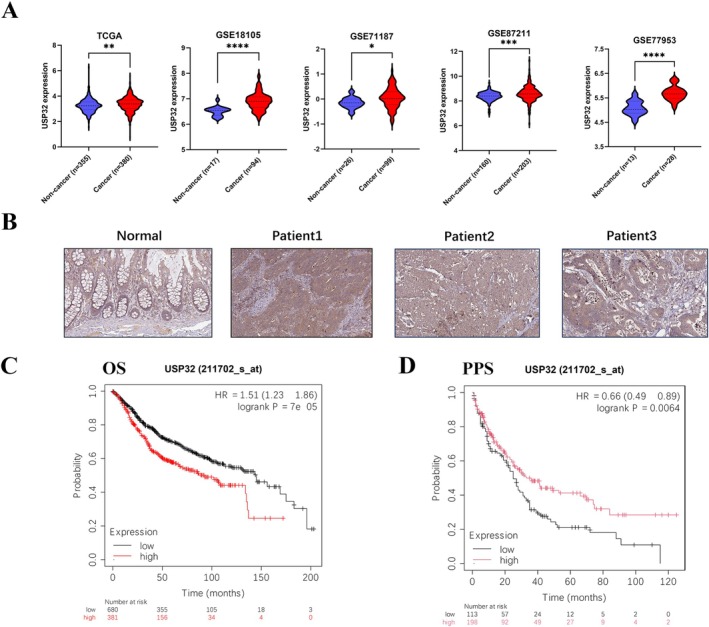
USP32 expression is markedly increased in CRC. (A) USP32 gene expression in CRC tumour and non‐cancer samples was obtained from TCGA‐COAD, GSE18105, GSE71187, GSE87211, and GSE77953. The Mann–Whitney‐Wilcoxon test was used. *p < 0.05, **p < 0.01, ***p < 0.001, ****p < 0.0001. (B) IHC staining of USP32 in CRC and normal colon samples was analysed by using the HPA database. (C) Kaplan–Meier analysis of the relationship between USP32 expression and OS of CRC patients. (D) Kaplan–Meier analysis of the relationship between USP32 expression and PPS of CRC patients.

The expression of USP32 in CRC was first investigated by analysing several public databases. In TCGA‐COAD, GSE18105, GSE71187, GSE87211, and GSE77953 datasets, USP32's gene expression was significantly higher in CRC tissues than in normal samples (*p* < 0.05) (Figure 1A). In addition, IHC staining shows that USP32 protein expression is relatively higher in cancer tissues compared with non‐cancer samples (Figure 1B). To investigate the clinical significance of USP32, we then performed the Kaplan–Meier plotter survival analysis of CRC patients. As a result, we found that high USP32 is significantly related to shorter overall survival (OS) of CRC patients (Figure 1C). In contrast, we discovered that high USP32 is significantly associated with longer post‐progression survival (PPS) of CRC patients (Figure 1D). The reasons for the different effects of USP32 on OS and PPS in CRC patients are still unclear.

### 3.2 USP32 Overexpression Is Related to NF‐κB Signalling Pathway Activation in CRC

**FIGURE 2:**
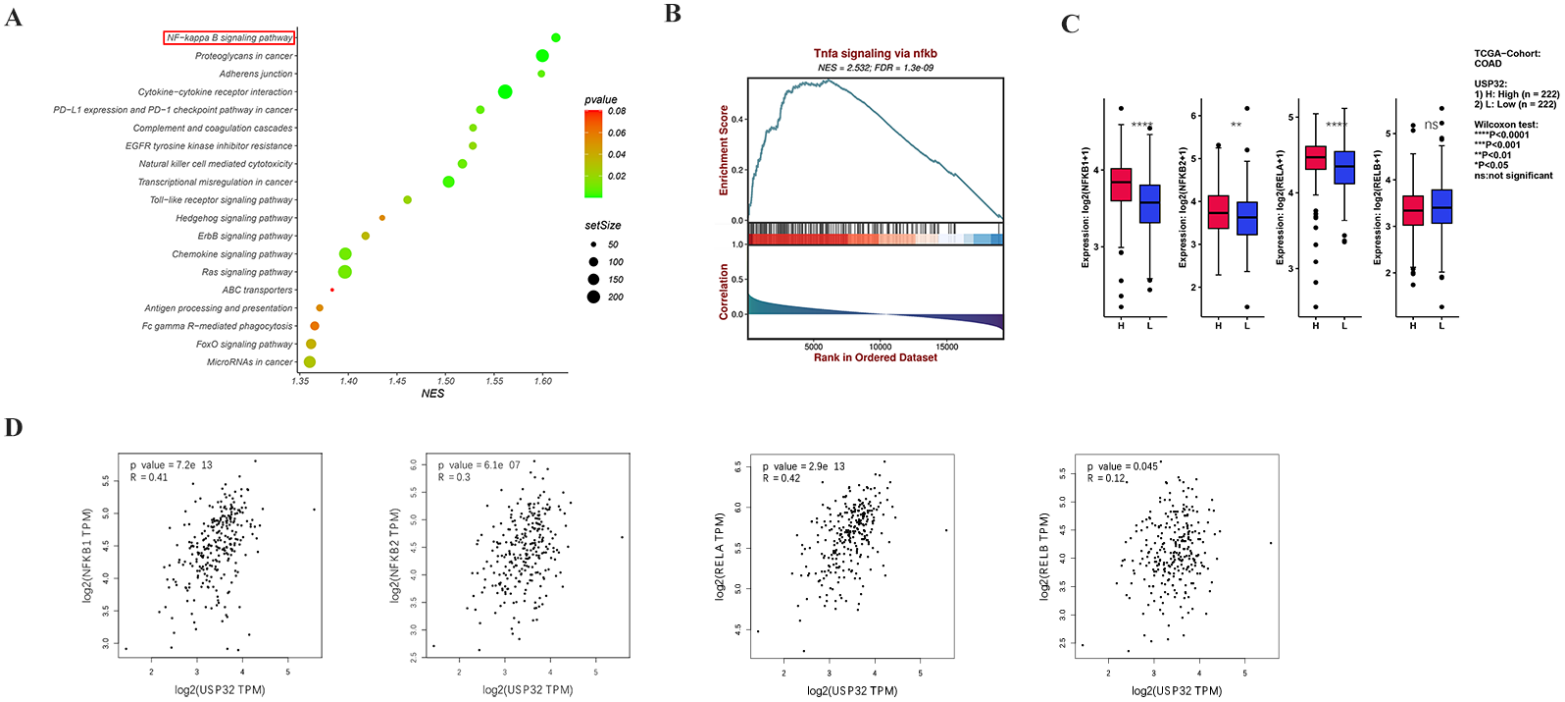
USP32 is related to NF‐κB activation in CRC. (A, B) GSEA analysis revealed that USP32 is mostly associated with NF‐κB activation in CRC. (C) The expression of NFKB1, NFKB2, RELA, and RELB in high‐ and low‐USP32 expression samples. The Mann–Whitney‐Wilcox test was used. (D) Correlation analysis of the expression of USP32 and NFKB1, NFKB2, RELA, and RELB in CRC.

To explore the potential mechanisms of USP32 involvement in CRC, we performed a GSEA analysis of 190 low‐ and 190 high‐USP32 expression CRC samples from the TCGA‐COAD database. As a result, we discovered that USP32's high expression is mostly related to the activation of the NF‐κB signalling pathway (NES score = 2.532, FDR = 1.3e‐09) (Figure 2A,B). In addition, the expression of key NF‐κB genes NFKB1, NFKB2, RELA and RELB is significantly higher in high‐USP32 expression samples compared with that in low‐USP32 expression samples (Figure 2C). Furthermore, correlation analysis shows that the expression of NFKB1 (*R* = 0.41, *p* = 7.2e‐13), NFKB2 (*R* = 0.3, *p* = 6.1e‐07), RELA (*R* = 0.42, *p* = 2.9e‐13) and RELB (*R* = 0.12, *p* = 0.045) is all markedly related to USP32's expression in CRC (Figure 2D).

### 3.3 USP32 Is Correlated With Immune Infiltrates in CRC

**FIGURE 3:**
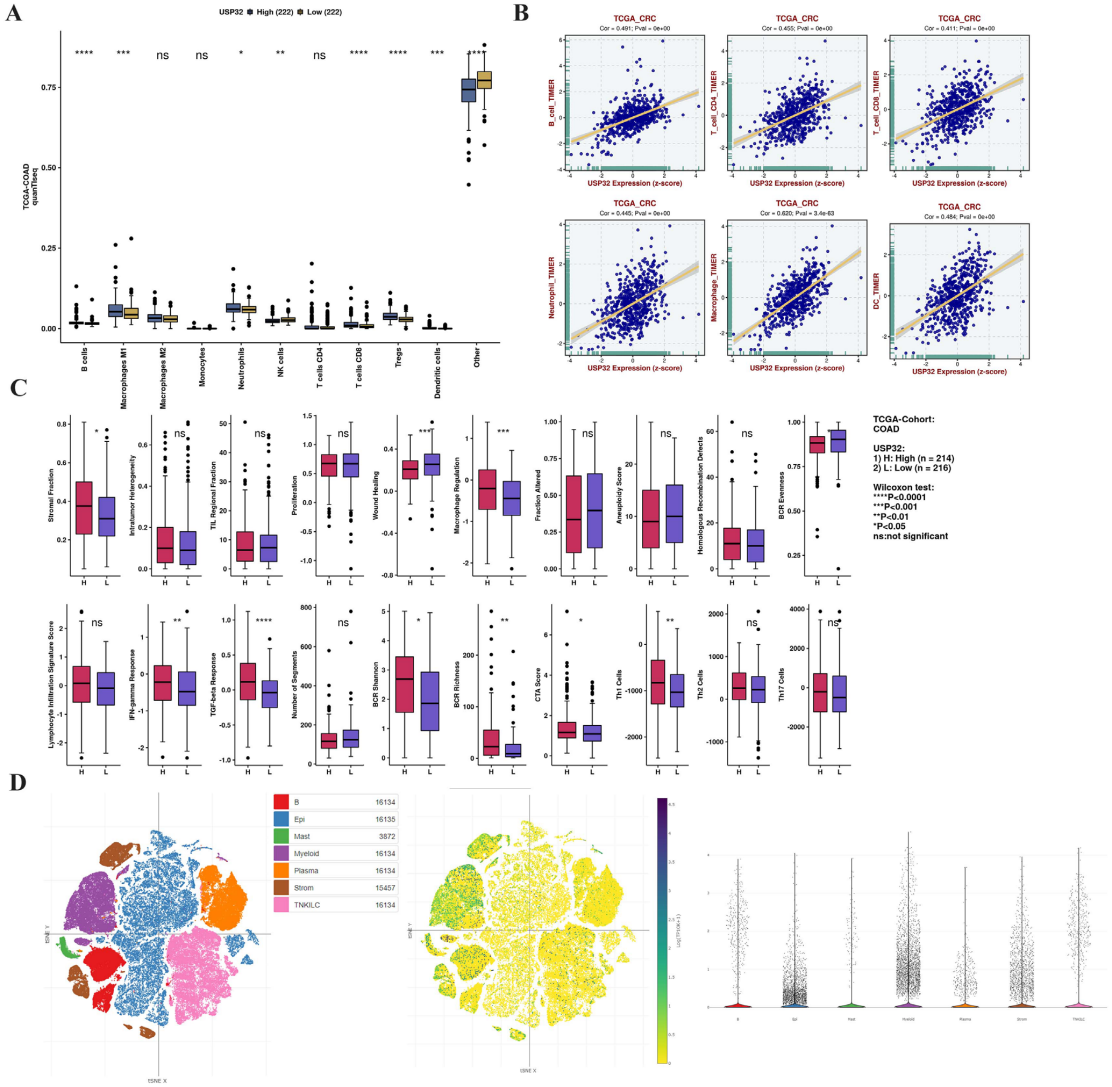
The relationship between USP32 and TME in CRC. (A) The fraction of immune infiltrates in high‐ and low‐USP32 expression CRC samples. (B) Correlation analysis of USP32 expression and the fraction of immune infiltrates in CRC. (C) The association between USP32 and immune scores in CRC. (D) The expression of USP32 in the TME of CRC.

To investigate the potential role of USP32 in the TME of CRC, we first used the quanTIseq algorithm to analyse the fraction of immune infiltrates in low‐ and high‐USP32 expression CRC samples. As a result, we revealed that the B cells, M1 macrophages, neutrophils, CD8+ T cells, Tregs and dendritic cells were markedly increased in the high‐USP32 expression CRC samples, while NK and other immune cells were markedly increased in the low‐USP32 expression CRC samples (*p* < 0.05) (Figure 3A). The TIMER algorithm also showed that USP32 expression is significantly associated with the fraction of B cells, CD4 and CD8 T cells, neutrophils, macrophages and dendritic cells (Figure 3B). Through using the CAMOIP database, we also revealed that USP32 expression is positively related to immune scores such as stromal fraction, macrophage regulation, IFN‐gamma response, TGF‐beta response, BCR Shannon, BCR richness and CTA score in CRC, while it is negatively related to immune scores such as wound healing and BCR evenness (Figure 3C). Finally, we investigated the expression of USP32 at single‐cell levels. As shown in Figure 3D, USP32 is expressed not only in tumour cells but also in B cells, mast cells, myeloid cells, plasma cells, stromal cells and TNKILC cells. These results indicate that USP32 may play a role in the TME of CRC.

### 3.4 USP32 Promotes CRC Progression Both In Vitro and In Vivo

**FIGURE 4:**
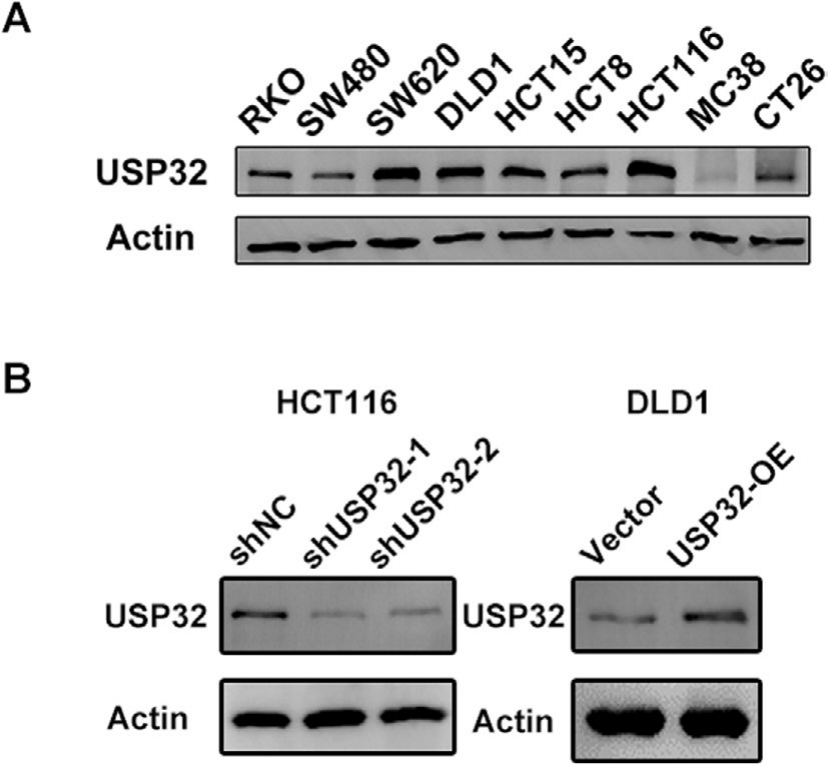
(A) The protein expression levels of USP32 in CRC cell lines. (B) Stable knockdown of USP32 in HCT116 cells and overexpression of USP32 in DLD1 cells.

**FIGURE 5:**
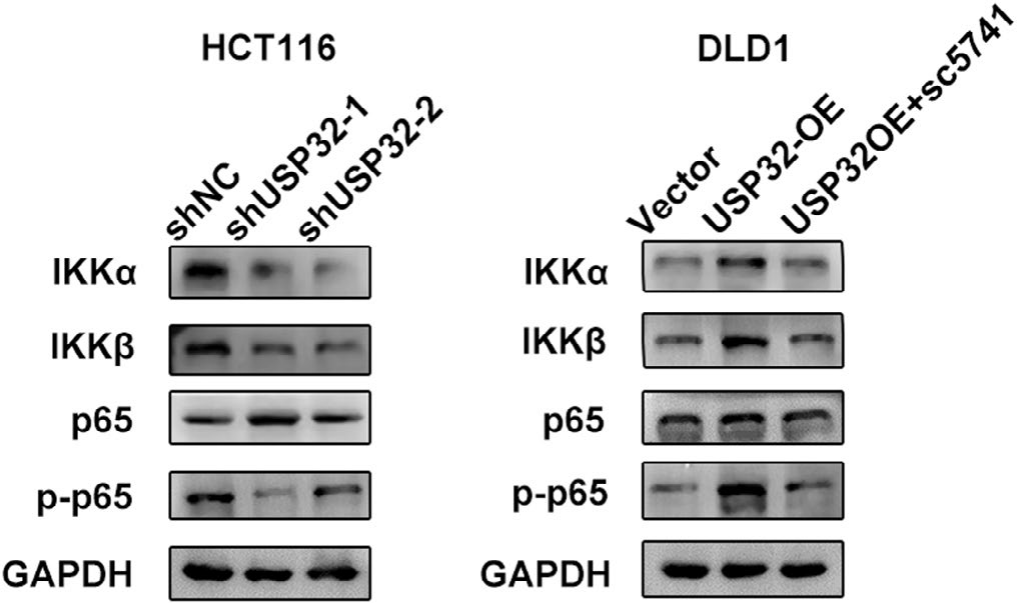
The effect of USP32 knockdown/overexpression with or without sc5731 treatment on the expression of key NF‐κB signalling pathway members in HCT116 and DLD1 cells.

**FIGURE 6:**
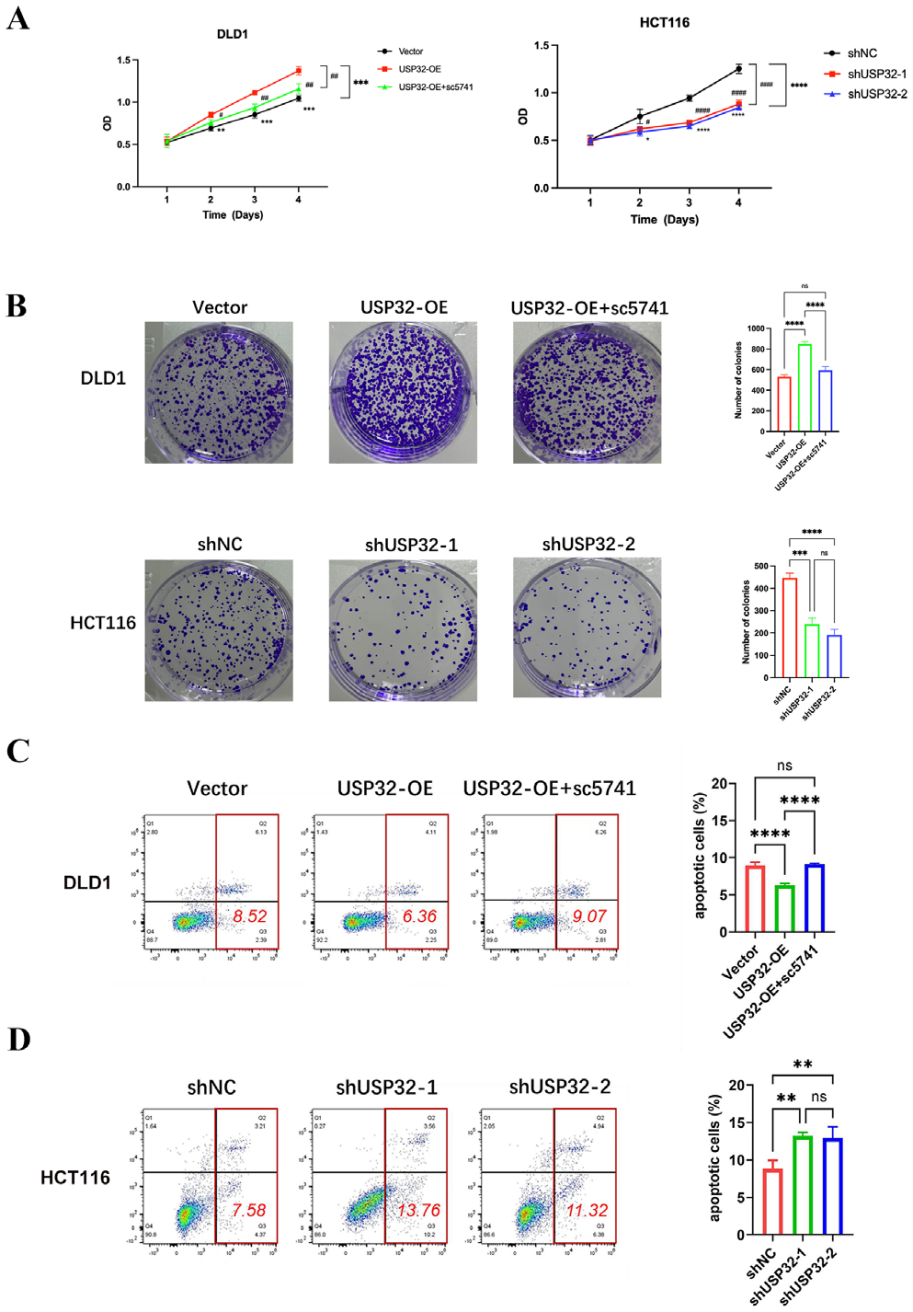
The effect of USP32 knockdown/overexpression with/without sc5731 treatment on the (A) proliferation and (B) colony formation, and (C and D) anti‐apoptosis ability of CRC cells.

**FIGURE 7:**
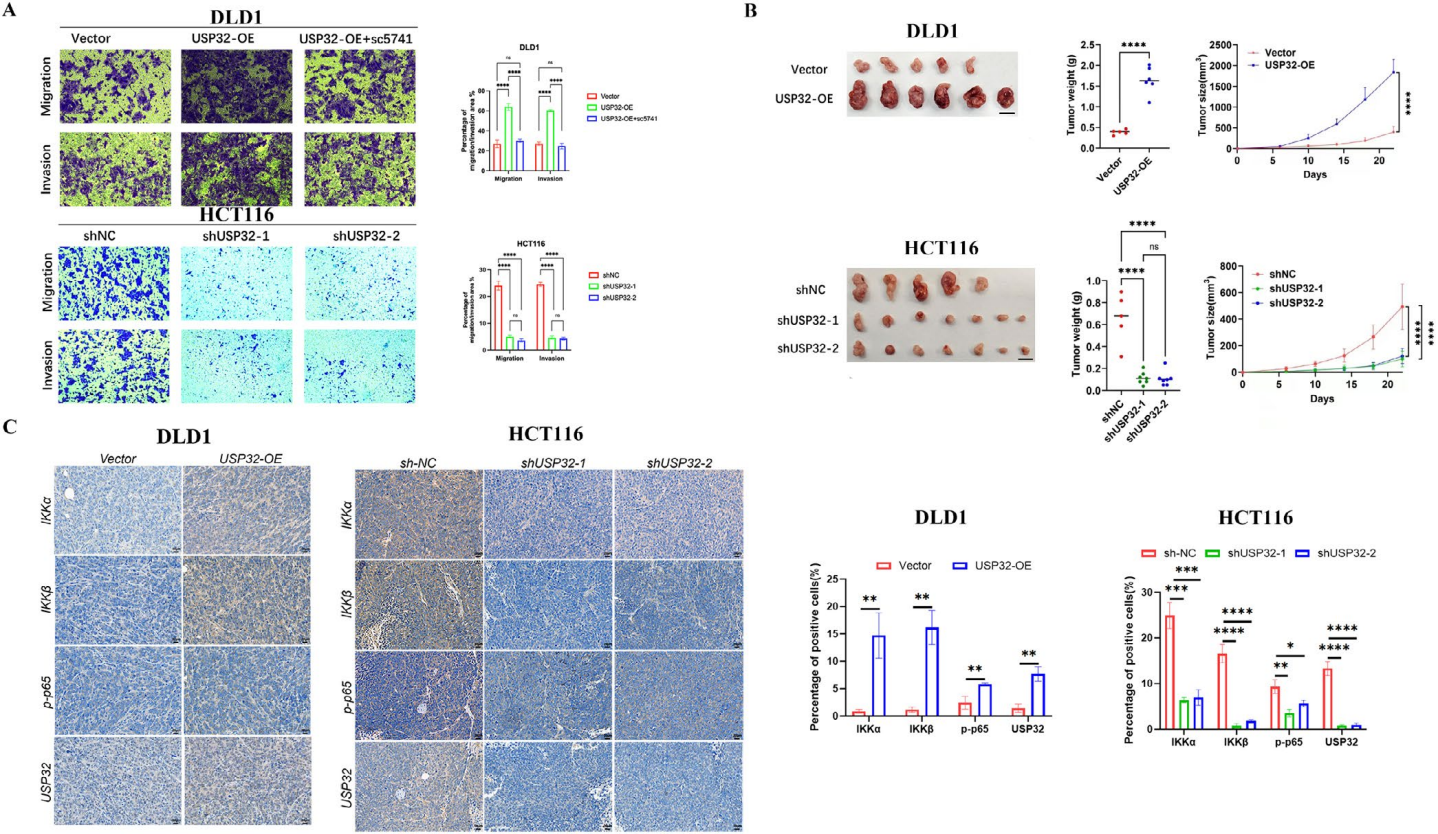
The effect of USP32 knockdown/overexpression with/without sc5731 treatment on (A) the migration ability of CRC cells in vitro and (B) the tumour growth of CRC cells in vivo. (C) IHC analysis of IKKα, IKKβ, p‐p65 and USP32 expression in DLD1 and HCT116 xenograft tumours.

To explore the potential roles of USP32 in CRC, we first detected the expression of USP32 in nine CRC cell lines (Figure 4A). Two CRC cell lines, DLD1 and HCT116, were then selected to construct USP32 overexpression and knockdown models, respectively. WB experiments confirmed the effective overexpression and knockdown of USP32 expression in DLD1 and HCT116 cells (Figure 4B). To investigate whether the NF‐κB signalling pathway is involved in the USP32‐derived development of CRC, an inhibitor of NF‐κB, named sc5741, was used to perform subsequent experiments. As a result, we found that the knockdown of USP32 expression could decrease the protein expression levels of IKKα, IKKβ and p‐p65, while USP32 overexpression could increase the protein expression levels of IKKα, IKKβ and *p*‐p65, which was abrogated upon sc5741 treatment (Figure 5). Functional in vitro experiments also confirmed that USP32 could support the proliferation, colony formation, anti‐apoptosis and migration abilities of CRC cell lines, which is supported by the NF‐κB signalling pathway (Figures 6 and 7). As for in vivo experiments, we first used a xenograft CRC nude mice model to verify USP32's oncogenic role in CRC. As shown in Figure 7A,B, USP32 overexpression could significantly increase CRC tumour weight and volume, while the knockdown of USP32 had opposite functions. In addition, IHC analysis of DLD1 and HCT116 xenograft tumours confirmed that USP32 could positively regulate the NF‐κB signalling pathway (Figure 7C). The above results show that USP32 promotes CRC progression via the activation of the NF‐κB signalling pathway.

To verify the role of USP32 in the TME of CRC, we then constructed CT26–USP32 overexpressing cells (Figure S1A). The results of in vivo studies showed that USP32 overexpression significantly promoted CT26 tumour progression (Figure S1B). As MDSCs and M2 macrophages have been reported to play key roles in repressing anti‐tumour immunity, we then performed flow cytometry analysis of CT26 tumours. As a result, we found that USP32 overexpression could markedly enhance the fractions of infiltrating MDSCs and M2 macrophages in CT26 tumours, indicating its role in repressing anti‐tumour immunity (Figure S1C,D).

## 4 Discussion

Several studies found that DUBs, especially USPs, have multiple functions during cancer progression [22, 23]. Although the roles of USP32 in cancer are widely reported, there are no studies that have focused on the role of USP32 in CRC. In our study, we first discovered the overexpression of USP32 in CRC. We also revealed that high USP32 expression is associated with poor prognosis in CRC patients.

TME plays a key role in CRC and significantly affects the response to immunotherapy of CRC patients [24, 25]. Through bioinformatics analyses, we found that USP32's high expression is associated with the higher fractions of several kinds of immunosuppressive cells such as B cells, Tregs, neutrophils and macrophages. B cells are reported to inhibit anti‐tumour T cell responses through antigen non‐specific mechanisms in CRC [26]. Tregs are widely discovered to negatively regulate T cells' anti‐tumour toxicity and induce immune escape responses [27, 28, 29]. Neutrophils are a fraction of cells that release neutrophil extracellular traps that drive CRC development and EMT [30, 31]. Tumour‐associated macrophages (TAMs), especially an M2 phenotype, generally have tumour‐promoting activities that induce metastasis and immunosuppression [32, 33].

To reveal the potential molecular mechanisms that USP32 may be involved in, we then performed GSEA analysis of high‐ and low‐USP32 expression CRC samples, and we found that USP32 activation is mostly associated with the activation of the NF‐κB signalling pathway. The NF‐κB family comprises RelA (p65), RelB, c‐Rel, p105/p50 and p100/p52 [34]. Upon receiving extracellular signals, IκBα undergoes phosphorylation by an inhibitor of κB kinase (IKK), followed by ubiquitination and proteasomal degradation [35]. Inversely, upon inactivation of the NF‐κB pathway, the inhibitor of κB protein (IκB) associates with p65/p50 heterodimers to sequester NF‐κB in the cytosol without nucleocytoplasmic shuttling [36]. Aberrant NF‐κB activation has been observed in both tumour epithelial cells and stromal myofibroblasts surrounding CRC [37, 38]. In CRC cells, NF‐κB signalling has been found to play multiple roles in regulating CRC progression. For example, NF‐κB signalling could promote the secretion of CCL20, thus recruiting Tregs to promote CRC chemoresistance [39]. In addition, NF‐κB signalling could also induce the secretion of CXCL1 and the recruitment of CXCR2 + MDSCs into CRC tumours, thus impairing effective T cell responses during anti‐PD1 therapy [40]. In stromal myofibroblasts, NF‐κB was found to induce the expression of COX‐2, which may have potential effects on adjacent cancer epithelial cells to promote CRC tumourigenesis and angiogenesis [38]. In the present study, we discovered that NF‐κB signalling is activated by USP32 overexpression and that the inhibition of the USP32–NF‐κB axis could reverse the proliferation, colony formation, anti‐apoptosis and migration abilities of CRC cells.

To further verify USP32's oncogenic roles, we also performed in vivo studies. We found that USP32 downregulation could suppress CRC tumour growth while overexpressing USP32 in CRC cells had the opposite function. The results of the IHC analysis confirmed that the expression of USP32 is critical for the activation of NF‐κB signalling. To further verify the role of USP32 in CRC tumour immunity, we also explored the positive regulatory relationship between USP32 expression and the infiltration of MDSC and M2 macrophage cells in CRC tumours.

However, our project also has several limitations. First, our hospital lacked enough CRC samples as a validation cohort for IHC analysis. We hope that future studies will perform IHC analysis of large‐sample cohorts to verify the relationship between USP32 and NF‐κB signalling. Second, USP32 primarily performs biological functions by mediating the de‐ubiquitination process, and the mechanism of how USP32 activates NF‐κB expression remains unexplored.

## 5 Conclusions

In conclusion, our study has found that USP32 promotes CRC development by activating the NF‐κB signalling pathway, and the USP32 –NF‐κB axis may act as a potential target for CRC therapy.

## Author Contributions

**Xiaofan Duan:** conceptualization (equal), data curation (equal), validation (equal), visualization (equal), writing – original draft (equal), writing – review and editing (equal). **Gaoshaer Yeerkenbieke:** conceptualization (equal), data curation (equal), formal analysis (equal), validation (equal), writing – original draft (equal), writing – review and editing (equal). **Siping Huang:** conceptualization (equal), data curation (equal), supervision (equal), visualization (equal). **Yanjun Feng:** funding acquisition (equal), investigation (equal).

## Conflicts of Interest

The authors declare no conflicts of interest.

## Supporting information


**Figure S1.** The effects of USP32 overexpression on the infiltration of MDSCs and M2 macrophages in CT26 tumours. (A) Stable overexpression of USP32 in CT26 cells. (B) USP32 overexpression could promote the growth of CT26 tumours. (C) USP32 overexpression could promote the infiltration of MDSCs in CT26 tumours. (D) USP32 overexpression could promote the infiltration of M2 macrophages in CT26 tumours.

## Data Availability

The datasets analysed during the current study are available in the TCGA‐COAD from the TCGA database and GSE18105, GSE71187, GSE87211, and GSE77953 from the Gene Expression Omnibus database.
