# Supplementary figures and images for: USP32 Promotes Colorectal Carcinoma Progression Through Activating NF‐κB Signalling Pathway
Source: J Cell Mol Med. 2025 Mar 23;29(6):e70457. doi: 10.1111/jcmm.70457 (PMC11930632; doi:10.1111/jcmm.70457)

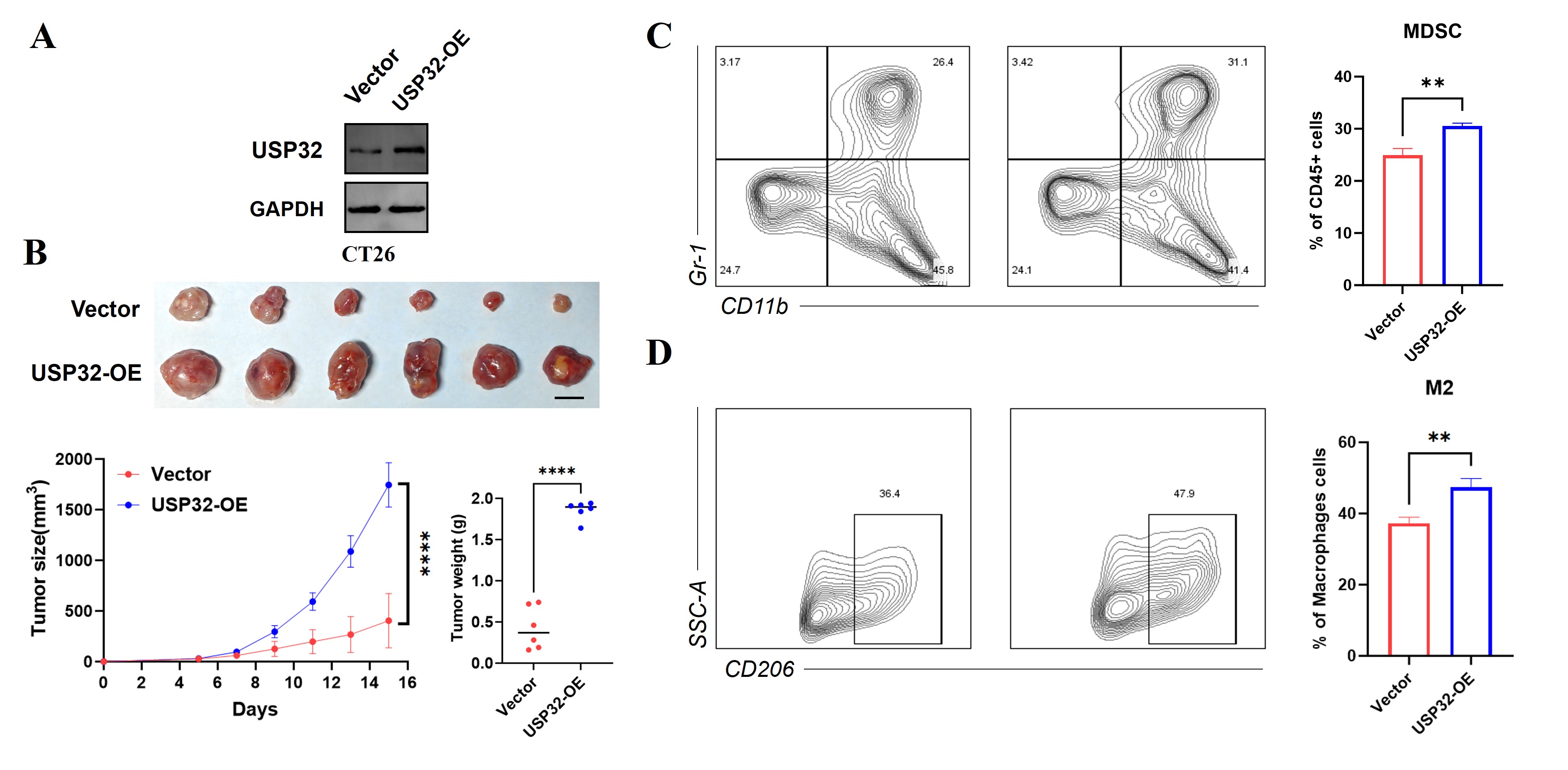

Supplement: Supplementary file 1 — Figure S1. The effects of USP32 overexpression on the infiltration of MDSCs and M2 macrophages in CT26 tumours. (A) Stable overexpression of USP32 in CT26 cells. (B) USP32 overexpression could promote the growth of CT26 tumours. (C) USP32 overexpression could promote the infiltration of MDSCs in CT26 tumours. (D) USP32 overexpression could promote the infiltration of M2 macrophages in CT26 tumours. [file JCMM-29-e70457-s001.jpg]
